# Two novel human coronavirus OC43 genotypes circulating in hospitalized children with pneumonia in China

**DOI:** 10.1080/22221751.2021.2019560

**Published:** 2022-01-04

**Authors:** Zhaoyong Zhang, Wenkuan Liu, Shengnan Zhang, Peilan Wei, Lu Zhang, Dehui Chen, Shuyan Qiu, Xiaobo Li, Jingxian Zhao, Yongxia Shi, Rong Zhou, Yanqun Wang, Jincun Zhao

**Affiliations:** a State Key Laboratory of Respiratory Disease, National Clinical Research Centre for Respiratory Disease, Guangzhou Institute of Respiratory Health, the First Affiliated Hospital of Guangzhou Medical University, Guangzhou, Guangdong, People’s Republic of China; b Guangzhou Customs District Technology Centre, Guangzhou, People’s Republic of China; c Institute of Infectious Disease, Guangzhou Eighth People’s Hospital of Guangzhou Medical University, Guangzhou, Guangdong, People’s Republic of China; d Guangzhou Laboratory, Bio-island, Guangzhou, Guangdong, People’s Republic of China

**Keywords:** HCoV-OC43, hospitalized children, pneumonia, novel genotype, recombinant

## Abstract

HCoV-OC43 is one of the mildly pathogenic coronaviruses with high infection rates in common population. Here, 43 HCoV-OC43 related cases with pneumonia were reported, corresponding genomes of HCoV-OC43 were obtained. Phylogenetic analyses based on complete genome, orf1ab and spike genes revealed that two novel genotypes of HCoV-OC43 have emerged in China. Obvious recombinant events also can be detected in the analysis of the evolutionary dynamics of novel HCoV-OC43 genotypes. Estimated divergence time analysis indicated that the two novel genotypes had apparently independent evolutionary routes. Efforts should be conducted for further investigation of genomic diversity and evolution analysis of mildly pathogenic coronaviruses.

## Introduction

There are four mildly pathogenic coronaviruses that can infect humans, HCoV-OC43, 229E, NL63 and HKU1, that mainly cause mild and self-limiting respiratory tract infections [1–4]. These mildly pathogenic coronaviruses were circulated endemically, contributing to episodes of human common cold worldwide and severe infection in immunosuppressed patients and infants [5,6]. HCoV-OC43 is the most prevalent HCoV in respiratory tract infections, which is believed to associate with fatal encephalitis [7]. Gene sequence of HCoV-OC43 showed a high similarity to bovine coronavirus and canine coronavirus [8]. 9-O-acetyl-sialic acid is believed to be the receptor to HCoV-OC43, which is the common receptor to Bovine CoV and HCoV-HKU1 [9].

Due to the capacity of coronaviruses to adapt to different hosts and make high-frequency mutations spontaneously in spike, it is easier for coronavirus to cross species barriers and enhance infectivity through successive generations [10]. Thus, it is urgently necessary to conduct virus genomic diversity and evolution investigation, especially for mildly pathogenic coronaviruses [11]. Here, a cluster of HCoV-OC43 related hospitalized children were enrolled in this study. The further genomic phylogenetic and evolution analysis of obtained sequences suggested two novel HCoV-OC43 genotypes (genotypes J and K) emerged in China. The discovery of novel HCoV-OC43 genotypes in human sheds light on the evolutionary trend and potential health threats of mildly pathogenic coronaviruses.

## Methods

A total of 43 HCoV-OC43-positive (RT-PCR positive) children with pneumonia hospitalized in the First Affiliated Hospital of Guangzhou Medical University between 2009 and 2021 were enrolled in this study. Viral RNAs were extracted from patients’ nasopharyngeal swab and preprocessed according to the official recommended sequencing protocol. Sequencing and data processing were using Illumina NextSeq 550 (Illumina, San Diego, MD, USA). Contigs and consensus sequences were obtained by using CLC Genomics Workbench v11.0 (Qiagen, Germantown, MD, USA) based on HCoV-OC43 prototype strain (Accession Number: NC_006213.1). Phylogenetic analyses based on complete genome, orf1ab and spike genes were performed and analyzed by using the Neighbor-Joining method with 1000 bootstrap replicates in MEGA 5.1 software. Estimated divergence time analysis of genotypes was performed by Bayesian Evolutionary Analysis by Sampling Trees (BEAST v1.7.5) program to determine the most recent common ancestor (tMRCA) of the novel genotypes based on spike gene. Estimation of pairwise genetic distances was performed and analyzed by using the Neighbor-Joining method with 1000 bootstrap replicates in MEGA 5.1 software. Bootscan analysis in SimPlot software was used for potential recombination events detection.

## Results

A cluster of 43 HCoV-OC43 infected children were enrolled in this study. The majority of patients who were infected with HCoV-OC43 had pneumonia, lobular pneumonia or acute bronchitis. Their ages ranged from 1.6 months to 8 years old. HCoV-OC43 RT-PCR positive samples were also tested for other 14 common respiratory pathogens, including HCoV-229E, HCoV-OC43, HCoV-HKU1, influenza A virus (Flu A), influenza B virus (Flu B), enterovirus (EV), human bocavirus (HBoV), human rhinovirus (HRV), respiratory syncytial virus (RSV), adenovirus (ADV), four types of human parainfluenza virus (HPIV), Mycoplasma pneumoniae (MP), human metapneumovirus (HMPV) and Chlamydia pneumonia (CP) [11]. Of the 43 patients, 26 (60.5%) of them were infected only with HCoV-OC43, while 17 (39.5%) patients were found to be co-infected with other pathogens (Supplementary Table 1). Only 45.2% (14/31) of subjects have settled in Guangzhou and others from all over the country.

**Figure 1.**
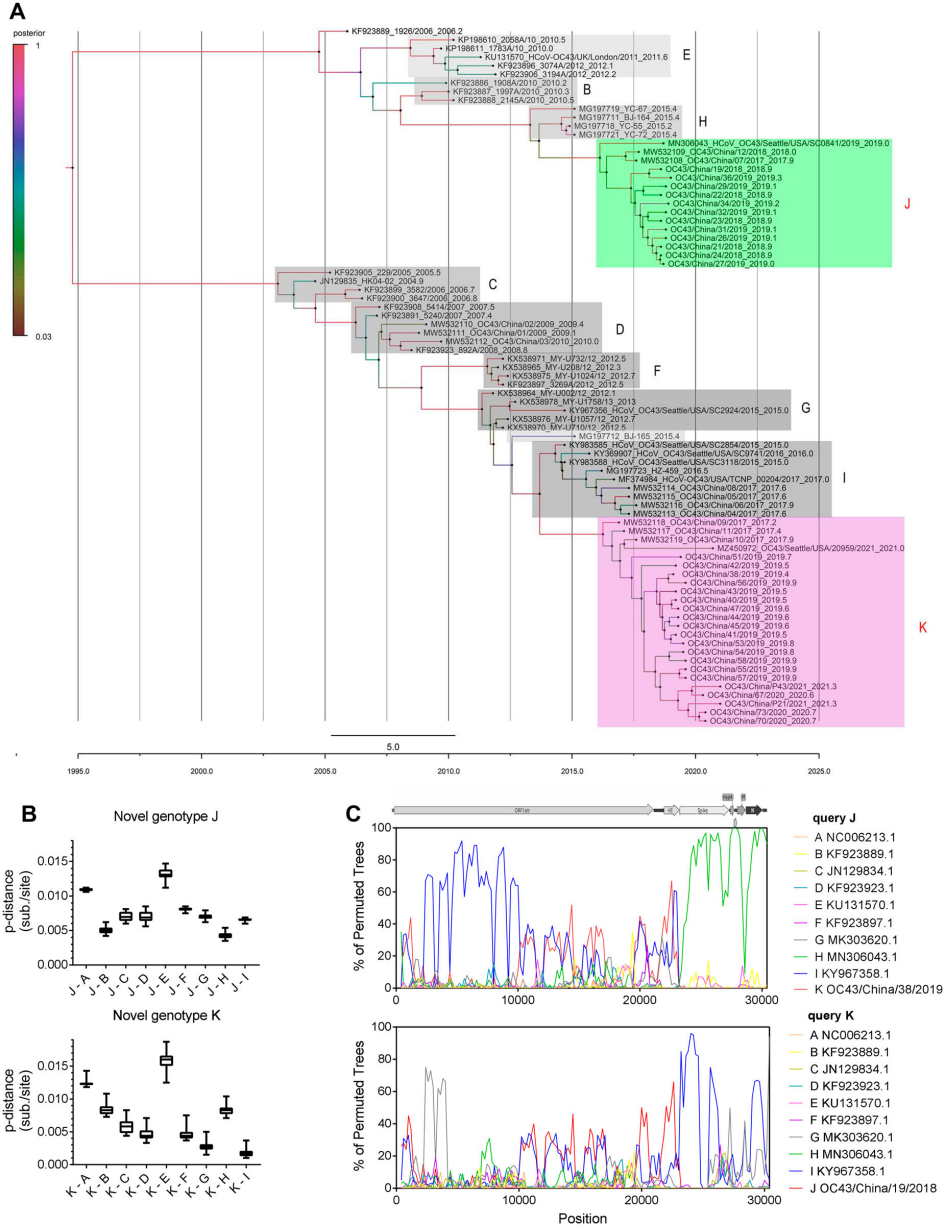
Analysis of time-resolved phylogeny, pairwise genetic distances and genetic recombination of two novel genotypes of HCoV-OC43. (A) Beast software was used to conduct estimated divergence time analysis of novel genotypes of HCoV-OC43 based on spike gene. A HKY nucleotide substitution model with estimated base frequencies and a lognormal relaxed clock model were used for evolution analysis. The Markov chain Monte Carlo (MCMC) analysis was performed with 1 × 109 generations with every 10,000 generations were sampled. The sampling year, strain name and accession number are shown on labels. Colour on branch represented the posterior probability corresponding to the legend. Two novel genotypes are indicated in green (J) and pink (K), respectively. (B) Estimation of pairwise genetic distances of novel genotypes J and K to previous genotypes based on the full-length genome sequences, respectively. (C) Genetic recombinant analyses of HCoV-OC43 novel genotypes J and K.

After deleting overrepresented sequences, 38 HCoV-OC43 genome sequences of representative genotypes from the NCBI database with 43 HCoV-OC43 strains enrolled in this study were processed for the investigation of genetic relationship. As shown in Figure 1(A), all available HCoV-OC43 strains on NCBI can be divided into nine genotypes (A to I genotypes). However, two novel genotypes can be revealed after enrolling the genome sequences we acquired. Two novel genotypes, named genotype J and genotype K, occupy relatively independent positions in the phylogenetic tree. The time to a most recent common ancestor for the two genotypes was estimated to be between 4 and 6 years before the identification in 2014–2016. Besides, it is obvious that genotypes J and K were circulating simultaneously in 2019, and genotype K has become the dominant genotype gradually (Supplementary Table 1).

As shown in Figure 1(B), the genetic distance of the genotype J compared with genotypes A, B, C, D, E, F, G, and I were >0.5% (0.005 substitution/site), Phylogenetic trees based on spike and orf1ab gene showed different topologies associated with genotype J (Supplementary Figure 1). Recombination analysis also revealed that genotype J comes from the recombination between genotypes H and I (Figure 1(C), Supplementary Figure 1), while genotype K may be derived from genotype I. Above all, two novel genotypes of HCoV-OC43 associated with pneumonia emerged in China and showed different evolutionary routes.

## Discussion

In this study, 43 sequences of HCoV-OC43 were enrolled for phylogenetic and evolution analysis, which revealed 2 novel genotypes circulating in China. HCoV-OC43 was circulated endemically, contributing to episodes of human common cold worldwide and severe infection in immunosuppressed patients and infants. However, the studies about HCoV-OC43 genomic diversity and evolution investigation are still rare due to the absence of enough genome sequence. There is no vaccine or specific treatment to protect against mildly pathogenic coronaviruses. Most people with mildly pathogenic human coronavirus illness will recover spontaneously, but people can have multiple infections in their lifetime. The discovery of two novel genotypes of HCoV-OC43 associated with pneumonia alarmed the significance of the monitoring common coronavirus variants.

## Ethics statement

Sample collection and all experiments in the present study were performed with Ethical Approval given by Ethics Committee of the First Affiliated Hospital of Guangzhou Medical University.

## Supplementary Material

Supplemental Material

## References

[1] Almeida JD, Tyrrell D. The morphology of three previously uncharacterized human respiratory viruses that grow in organ culture. J Gen Virol. 1967;1:175–178. 4293939 10.1099/0022-1317-1-2-175

[2] Kapikian AZ, James Jr HD, Kelly SJ, et al. Isolation from man of “avian infectious bronchitis virus-like” viruses (coronaviruses) similar to 229E virus, with some epidemiological observations. J Infect Dis. 1969;119:282–290. 4976345 10.1093/infdis/119.3.282 PMC7110032

[3] van der Hoek L, Pyrc K, Jebbink MF, et al. Identification of a new human coronavirus. Nat Med. 2004;10:368. 15034574 10.1038/nm1024 PMC7095789

[4] Woo PC, Lau SK, Chu C-m, et al. Characterization and complete genome sequence of a novel coronavirus, coronavirus HKU1, from patients with pneumonia. J Virol. 2005;79:884–895. 15613317 10.1128/JVI.79.2.884-895.2005 PMC538593

[5] Annan A, Ebach F, Corman VM, et al. Similar virus spectra and seasonality in paediatric patients with acute respiratory disease, Ghana and Germany. Clin Microbiol Infect. 2016;22:340–346. 26585774 10.1016/j.cmi.2015.11.002 PMC7172147

[6] Konca C, Korukluoglu G, Tekin M, et al. The first infant death associated with human coronavirus NL63 infection. Pediatr Infect Dis J. 2017;36:231–233. 28081049 10.1097/INF.0000000000001390

[7] Morfopoulou S, Brown JR, Davies EG, et al. Human coronavirus OC43 associated with Fatal encephalitis. N Engl J Med. 2016;375:497–8. 27518687 10.1056/NEJMc1509458

[8] Szczepanski A, Owczarek K, Bzowska M, et al. Canine respiratory coronavirus, bovine coronavirus, and human coronavirus OC43: receptors and attachment factors. Viruses. 2019;11:328. 10.3390/v11040328 PMC6521053 30959796

[9] Hulswit RJG, Lang Y, Bakkers MJG, et al. Human coronaviruses OC43 and HKU1 bind to 9-O-acetylated sialic acids via a conserved receptor-binding site in spike protein domain A. Proc Natl Acad Sci U S A. 2019;116:2681–2690. 30679277 10.1073/pnas.1809667116 PMC6377473

[10] Luo CM, Wang N, Yang XL, et al. Discovery of novel bat coronaviruses in South China that use the same receptor as Middle East respiratory syndrome coronavirus. J Virol. 2018;92:e00116–18. 29669833 10.1128/JVI.00116-18 PMC6002729

[11] Wang Y, Li X, Liu W, et al. Discovery of a subgenotype of human coronavirus NL63 associated with severe lower respiratory tract infection in China, 2018. Emerg Microbes Infect. 2020;9:246–255. 31996093 10.1080/22221751.2020.1717999 PMC7034077

