# Supplementary material for: Two novel human coronavirus OC43 genotypes circulating in hospitalized children with pneumonia in China
Source: Emerg Microbes Infect. 2022 Jan 4;11(1):168–71. doi: 10.1080/22221751.2021.2019560 (PMC8741245; doi:10.1080/22221751.2021.2019560)
Supplement: Supplemental Material [file TEMI_A_2019560_SM7144.docx]

Supplement Table 1. The demographic and clinical profiles of enrolled patients and related information

| Demographic profile | | Length of Stay (Day) | Residence | Co-infection^#^ | Diagnosis | Strain Name | Accession No. | Collection Date |
| --- | --- | --- | --- | --- | --- | --- | --- | --- |
| Sampling age | Gender* |  |  |  |  |  |  |  |
| 2 | M | 7 | Beijing | RSV | acute bronchitis | OC43/China/01/2009 | MW532111 | 2009 |
| 5 | M | 5 | Beijing | - | acute bronchitis | OC43/China/02/2009 | MW532110 | 2009 |
| 3.5 | F | 8 | Beijing | - | acute bronchitis | OC43/China/03/2010 | MW532112 | 2010 |
| 2.4 | M | 4 | na | RSV | pneumonia | OC43/China/04/2017 | MW532113 | 2017 |
| 1.8 | F | 9 | na | - | lobular pneumonia | OC43/China/05/2017 | MW532115 | 2017 |
| 2.6 | F | 6 | na | - | lobular pneumonia | OC43/China/06/2017 | MW532116 | 2017 |
| 3 | M | 7 | na | - | pneumonia | OC43/China/07/2017 | MW532108 | 2017 |
| 2 | F | 5 | na | ADV | lobular pneumonia | OC43/China/08/2017 | MW532114 | 2017 |
| 1.5 | F | 7 | na | - | lobular pneumonia | OC43/China/09/2017 | MW532118 | 2017 |
| 2.5 | M | 6 | na | ADV | pneumonia | OC43/China/10/2017 | MW532119 | 2017 |
| 3 | F | 4 | na | - | acute bronchitis | OC43/China/11/2017 | MW532117 | 2017 |
| 2.8 | F | 7 | na | - | acute bronchitis | OC43/China/12/2017 | MW532109 | 2017 |
| 0.92 | M | 16 | Guangzhou | - | lobular pneumonia | OC43/China/19/2018 | OK318939 | 2018 |
| 1.67 | M | 6 | Guangzhou | - | pneumonia | OC43/China/21/2018 | OK318940 | 2018 |
| 3 | F | 23 | Beijing | - | pneumonia | OC43/China/22/2018 | OK318941 | 2018 |
| 1 | M | 9 | Zhaoqing | - | acute bronchitis | OC43/China/23/2018 | OK391221 | 2018 |
| 5 | F | 6 | Guangzhou | MP | lobular pneumonia | OC43/China/24/2018 | OK318942 | 2018 |
| 3 | F | 5 | Guangzhou | - | lobular pneumonia | OC43/China/26/2019 | OK391222 | 2019 |
| 2.75 | M | 4 | Guangzhou | MP | lobular pneumonia | OC43/China/29/2019 | OK391223 | 2019 |
| 1.42 | M | 6 | Hezhou | MP | lobular pneumonia | OC43/China/31/2019 | OK391224 | 2019 |
| 0.33 | M | 4 | Zengcheng | - | lobular pneumonia | OC43/China/32/2019 | OK391225 | 2019 |
| 4 | M | 4 | Yangjiang | - | lobular pneumonia | OC43/China/34/2019 | OK318944 | 2019 |
| 1.42 | F | 5 | Meizhou | MP | pneumonia | OC43/China/36/2019 | OK391226 | 2019 |
| 0.14 | F | 4 | Guangzhou | - | bronchopneumonia | OC43/China/38/2019 | OK318918 | 2019 |
| 8 | F | 4 | Guangzhou | MP | lobular pneumonia | OC43/China/40/2019 | OK318945 | 2019 |
| 0.17 | F | 4 | Guangzhou | CT | lobular pneumonia | OC43/China/41/2019 | OK391238 | 2019 |
| 2 | M | 9 | Guangzhou | ADV, MP | lobular pneumonia | OC43/China/42/2019 | OK391231-37 | 2019 |
| 2.5 | F | 5 | Guangzhou | - | bronchitis | OC43/China/43/2019 | OK391227 | 2019 |
| 0.33 | M | 4 | Conghua | MP, CMV | lobular pneumonia | OC43/China/44/2019 | OK391229 | 2019 |
| 1.83 | M | 3 | Zengcheng | - | acute bronchitis | OC43/China/45/2019 | OK318946 | 2019 |
| 1.67 | M | 4 | Qingyuan | - | lobular pneumonia | OC43/China/47/2019 | OK318917 | 2019 |
| 3.5 | M | 6 | Foshan | MP | pneumonia | OC43/China/51/2019 | OK318947 | 2019 |
| 1.42 | M | 5 | Ganzhou | MP, MRSA | bronchitis | OC43/China/53/2019 | OK500297 | 2019 |
| 2 | M | 6 | Guangzhou | ADV | severe pneumonia | OC43/China/54/2019 | OK500298 | 2019 |
| 1 | M | 6 | Zengcheng | - | lobular pneumonia | OC43/China/55/2019 | OK500299 | 2019 |
| 3 | F | 4 | na | MP | pneumonia | OC43/China/56/2019 | OK500300 | 2019 |
| 8 | M | 5 | Dongguan | - | pneumonia | OC43/China/57/2019 | OK500301 | 2019 |
| 4.83 | F | 4 | Guangzhou | - | lobular pneumonia | OC43/China/58/2019 | OK500302 | 2019 |
| 7 | F | 12 | Guangzhou | MP | lobular pneumonia | OC43/China/67/2019 | OK500303 | 2019 |
| 2.33 | M | 14 | ZhanJiang | - | lobular pneumonia | OC43/China/70/2019 | OK500304 | 2019 |
| 3.58 | M | 11 | Guangzhou | - | bronchitis | OC43/China/73/2019 | OK500305 | 2019 |
| 2.5 | F | 94 | na | - | pneumonia | OC43/China/P21/2021 | OK391228 | 2021 |
| 4 | F | 4 | na | - | pneumonia | OC43/China/P43/2021 | OK391230 | 2021 |


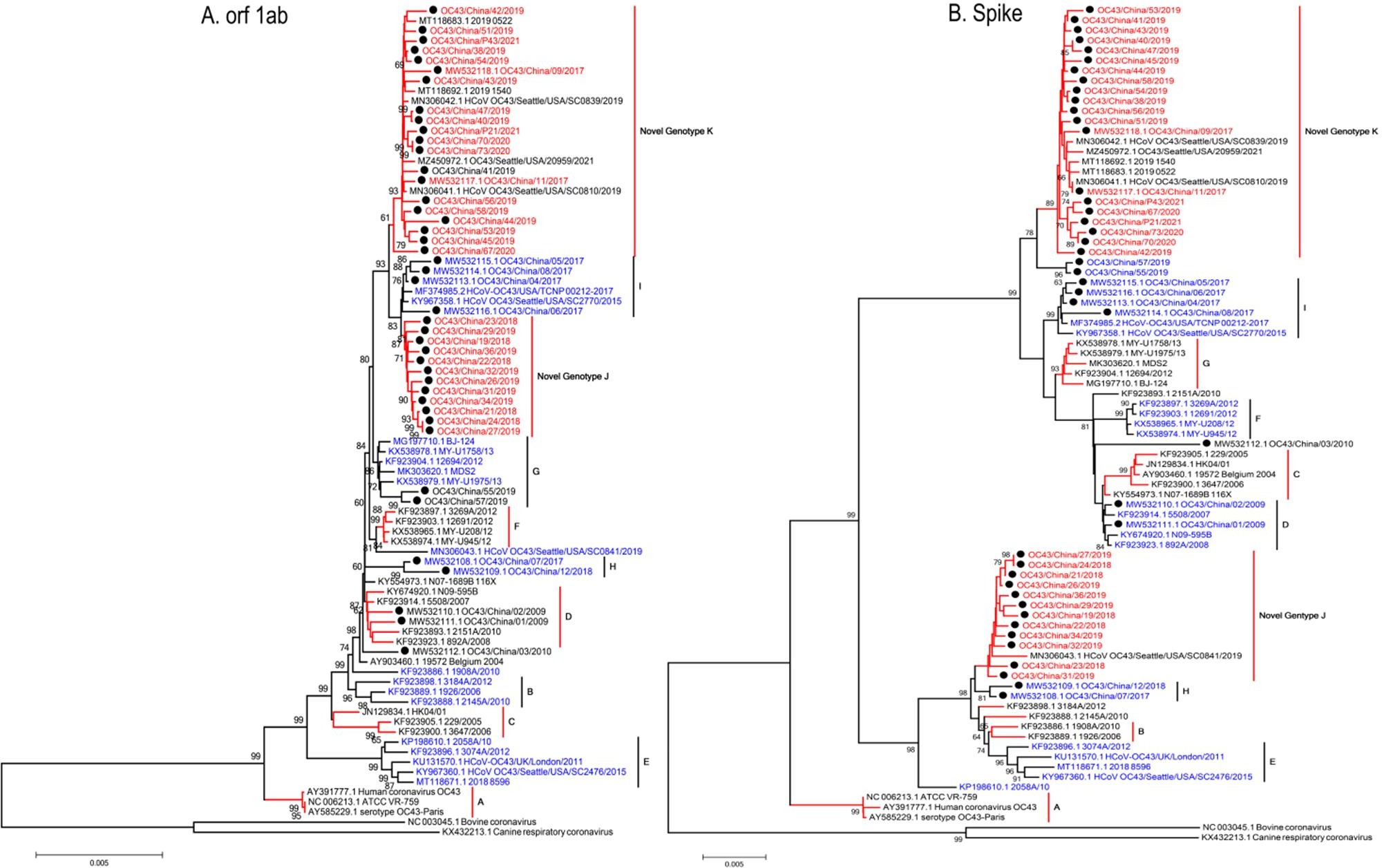
Supplement Figure 1. Phylogenetic analysis based on orf1ab and spike genes of HCoV- OC43
